# The Nonsubsampled Contourlet Transform Based Statistical Medical Image Fusion Using Generalized Gaussian Density

**DOI:** 10.1155/2015/262819

**Published:** 2015-10-07

**Authors:** Guocheng Yang, Meiling Li, Leiting Chen, Jie Yu

**Affiliations:** ^1^School of Computer Science and Engineering, University of Electronic Science and Technology of China, Chengdu 611731, China; ^2^Department of Biomedical Engineering, Sichuan Medical University, Zhongshan Road, Luzhou, Sichuan 646000, China; ^3^Provincial Key Laboratory of Digital Media, Chengdu 611731, China; ^4^School of Life Science and Technology, University of Electronic Science and Technology of China, Chengdu 610054, China

## Abstract

We propose a novel medical image fusion scheme based on the statistical dependencies between coefficients in the nonsubsampled contourlet transform (NSCT) domain, in which the probability density function of the NSCT coefficients is concisely fitted using generalized Gaussian density (GGD), as well as the similarity measurement of two subbands is accurately computed by Jensen-Shannon divergence of two GGDs. To preserve more useful information from source images, the new fusion rules are developed to combine the subbands with the varied frequencies. That is, the low frequency subbands are fused by utilizing two activity measures based on the regional standard deviation and Shannon entropy and the high frequency subbands are merged together via weight maps which are determined by the saliency values of pixels. The experimental results demonstrate that the proposed method significantly outperforms the conventional NSCT based medical image fusion approaches in both visual perception and evaluation indices.

## 1. Introduction

Multimodal medical image fusion (MIF) is a process of extracting complementary information from different source images and integrating them into a resultant image. The integration of multimodality medical images can provide more comprehensive pathological information for doctors, which greatly helps their diagnosis and treatment. For example, the fusion of computed tomography (CT) and magnetic resonance imaging (MRI) may simultaneously provide dense structures like bones and pathological soft tissue information. The combination of single-photon emission computed tomography (SPECT) and MRI image not only displays anatomical information, but also provides functional and metabolic information. Additionally, combining CT and the positive electron tomography (PET) image can concurrently visualize anatomical and physiological characteristics of the human body, the result of which is used to view tumor activity in oncology and discern tumor boundaries in organ diagnosis. Therefore, MIF technique can effectively provide support for medical diagnostic and healthcare.

Nowadays, multiresolution decomposition (MSD) based MIF has been recognized as an effective work, which can extract more abundant information from source images of different modalities. This technique has had a fast development and extensive application in the past decades. For example, Qu et al. [1] have utilized wavelet transform to fuse medical images. Ali et al. performed the combination of CT and MRI by the curvelet transform in [2] and Yang et al. proposed a fusion algorithm for multimodal medical images based on contourlet transform (CT) [3]. Li and Wang employed the nonsubsampled contourlet transform (NSCT) for the combination of MRI and SPECT in [4]. Compared with other multiscale decomposition, NSCT proposed by da Cunha et al. [5] is a more prominent tool and it has been successfully used in image denoising [6] and image enhancement [7]. Because of its properties of multiscale, multidirection, and the full shift-invariance, when it is used for image decomposition, it can capture the higher dimensional singularities such as edges and contours that cannot be effectively represented by the wavelets and avoid pseudo-Gibbs phenomena that presents in the contourlet transform. Specifically, when it is used for image fusion, the impacts of misregistration on the fused results can also be reduced effectively [8] and the correspondence between different subbands is easily found. Therefore, NSCT is more suitable for medical image fusion. Although medical image fusion methods based NSCT have achieved good results [9–13], most existing fusion methods neglect the dependencies between subband coefficients at the interscale and intrascale. However, the dependencies between decomposition coefficients commonly exist. What's more, the characteristics show non-Gaussian distribution and have the heavy tailed phenomenon. Thus making full use of the statistical dependencies between subband coefficients will effectively improve fusion performance.

**Figure 1:**
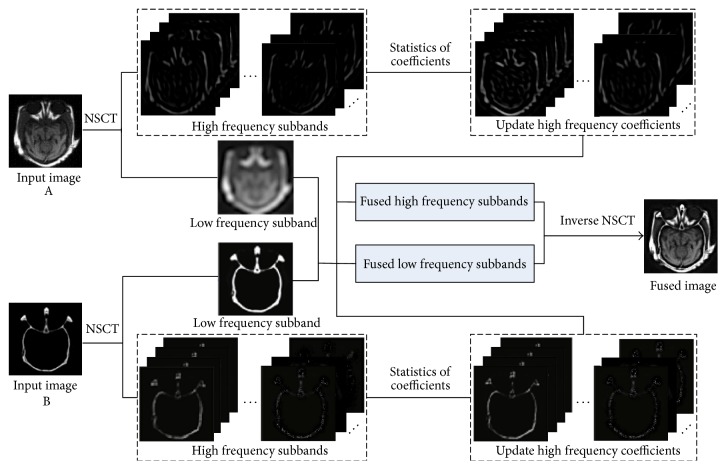
The schematic diagram of the proposed medical image fusion method.

In this paper, we present a novel NSCT based statistical multimodal MIF scheme, which utilizes generalized Gaussian density (GGD) to fit the marginal distributions of the high frequency coefficients and quantify the similarity measurement between two subbands by the symmetric Jensen-Shannon divergence (JSD) [14, 15] of two GGDs. Combining the relationships between subband coefficients, the high frequency coefficients are updated and finally fused. The general framework is shown in Figure 1. The main contributions of the proposed method are summarized as follows:

1. This study proposes a novel MIF method, which explores the dependencies between subband coefficients in NSCT domain.
2. GGD and JSD based statistical model is developed to nicely fit marginal distributions of the NSCT subband coefficients.
3. The new fusion rules are developed to fuse coefficients with the low frequency and high frequency, respectively.

The rest of this paper is organized as follows. In Section 2, related studies are reviewed. In Section 3, we first give a brief introduction of NSCT and then analyze the characteristics between subband coefficients in NSCT domain and compute their dependencies. The novel fusion rules are developed to fuse the low frequency subbands and high frequency subbands in Section 4.  Section 5 provides experimental results and discussion. Conclusions are drawn in the last section.

## 2. Related Research

A plethora of MIF methods based on NSCT assume that the coefficients of decomposition subbands are statistically independent; namely, there are no dependencies between subband coefficients across scales and within scale. Thus this kind of methods usually results in loss of some information of the source images. However, for a decomposition image using NSCT, there really exist the dependencies between subbands in different levels and different orientations at the same scale. Several famous statistical models based on multiresolution analysis have been proposed to characterize the dependencies of subband coefficients across scales. For example, the statistical models integrating Hidden Markov Tree (HMT) with the discrete wavelet transform (DWT) or the contourlet transform have been applied in the image denosing [16–18]. Moreover, the model of combining HMT and DWT is successfully applied in image segmentation [19]. As two recent examples, Wang et al. proposed two statistical models in the shift invariant shearlet transform domain, one combines the HMT [20] and the other utilizes GGD [21]. Although the statistical model based on HMT has successful applications, it contains some defects such as the low fitting precision, the high dependency for convergence of function, the lack of flexibility for the quad-tree structure itself, and so on.

In this paper, we present a novel statistical model to measure the dependencies of subband coefficients in NSCT domain. The advantage of the model is that one parent node may have any number of child leaves, instead of having limitation of one to four as HMT model. Our work seemingly shares some themes with literatures [21, 22], where the probability density function (PDF) of each decomposition subband is modeled with the GGD, and the similarity measurement between subbands is computed by the Kullback-Leibler distance (KLD) of two GGDs. However, our statistical model focuses on the statistics of the NSCT coefficients, and we evaluate the similarity of subbands across scales by the JSD rather than KLD. In addition, different fusion rules are, respectively, developed to combine components with low frequency and high frequency.

## 3. The Proposed Algorithm

### 3.1. Overview of NSCT

**Figure 2:**
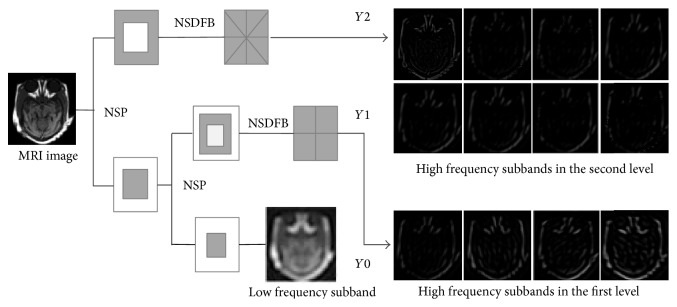
An illustration of the NSCT: the decomposition levels correspond to the first and second level and the number of directions of NSDFB is set to [2, 3], respectively.

NSCT, as a shift invariant version of contourlet, is an overcomplete transform with flexible multiscale, multidirectional expansion for images [5]. The decomposition process of the NSCT is divided into two phases, that is, the nonsubsampled pyramids (NSP) and the nonsubsampled directional filter bank (NSDFB). The former performs multiscale decomposition and the later provides direction decomposition. The NSP divides image into a low frequency subband and a high frequency subband in each level. Given that the decomposition level is *k*, NSP will generate *k* + 1 subband images, which consist of one low frequency image and *k* high frequency images. The subsequent NSDFB decomposes the high frequency subbands from NSP in each level. As for a specific subband, let the number of decomposition directions be *l*; then 2^*l*^ directional subbands are obtained, whose sizes are all the same as the source image. After the low frequency component is decomposed iteratively by the same way, an image is finally decomposed into one low frequency subimage and a series of high frequency directional subband images (∑_*j*=1_^*k*^2^*l*_*j*_^), wherein *l*_*j*_ denotes the number of decomposition directions at the *j* scale.  Figure 2 shows an intuitive example of NSCT. The diagram only enumerates the first two decomposition levels and the number of NSDFB directions is set to [2, 3] from coarser to finer scale.

### 3.2. Characteristics of the NSCT Subband Coefficients

**Figure 3:**
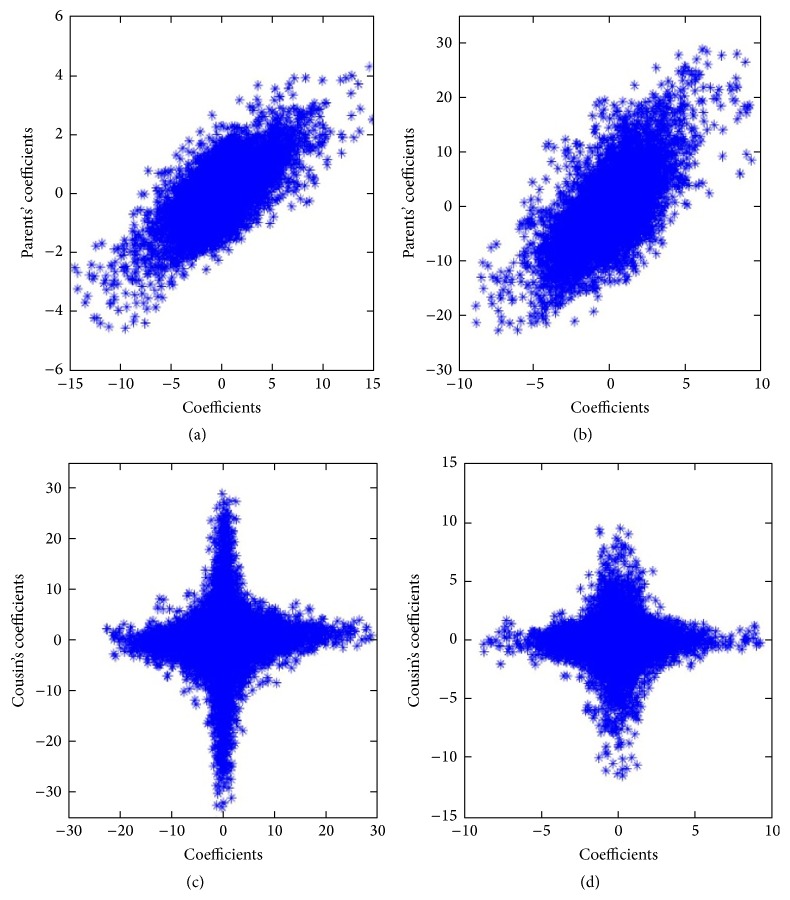
The conditional distribution between subband coefficients of the MRI image in Figure 2: (a) and (b) are the distribution characteristics between subband coefficients at different scales; (c) and (d) are distribution characteristics of subband coefficients with different directions at the same scale.

Figure 3 plots the conditional distributions of the NSCT coefficients, which characterizes the correlations between subband coefficients of the MRI image in Figure 2, wherein Figures 3(a) and 3(b) are probability distribution between two subband coefficients at different scales and Figures 3(c) and 3(d) are probability distribution between two subband coefficients with different directions at the same scale. Mathematically, the conditional distributions can be described as *P*(*X*∣*PX* = *px*) and *P*(*X*∣*CX* = *cx*); here, *px* and *cx* show the coefficients of parents and cousins. As shown in Figure 3, the relationships between subband coefficients demonstrate the nonlinear and interlaced aliasing on the whole, which illustrates that there exist interdependencies between subband coefficients in NSCT domain. Simultaneously, there is approximately independent or the slight correlation between subband coefficients with different directions at the same scale (cousin-cousin), while there is stronger correlation between subband coefficients at different scales (parents-children). Thus, the relationships of the NSCT coefficients mainly exist between parents and children.

**Figure 4:**
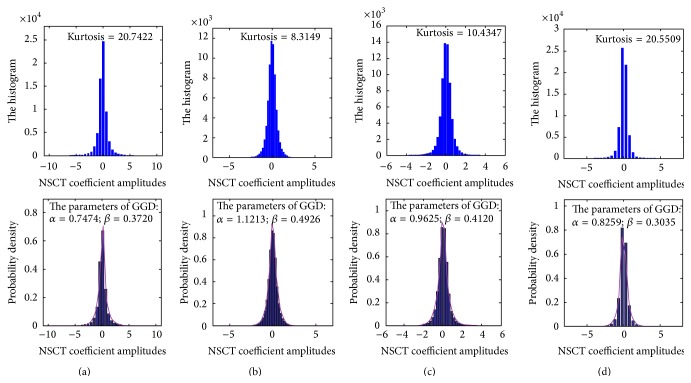
Histograms of four distribution maps in Figure 3 (the first row) and the curves fitted with GGDs (the second row).

Figure 4 corresponds to the histograms of four subimages in Figure 3. Obviously, all the characteristic diagrams have similar features with a very sharp peak at the zero amplitude and the extended tails in both sides of the peak, which indicates that the NSCT coefficients are sparse and the majority of coefficients are close to zero. Further, the kurtosis of each map is, respectively, measured as 20.74, 8.32, 10.44, and 20.55 (corresponding to (a), (b), (c), and (d) of the first row of Figure 4 in order). Clearly, these values are much larger than the kurtosis of Gaussian distribution (kurtosis is about 3.0). What is more, through a large number of experiments, the coefficient characteristics (sparse and heavy tailed phenomenon) are similar for other NSCT subbands. So there exists a fact that the NSCT coefficients are sparse and highly non-Gaussian.

**Figure 5:**
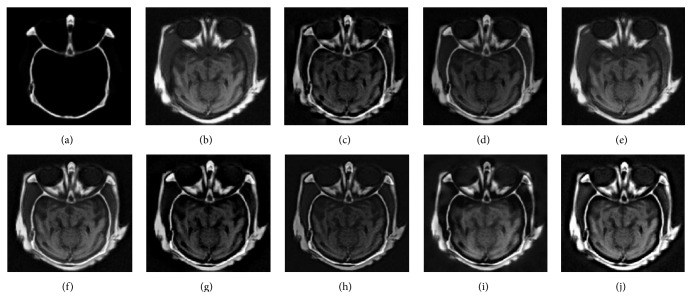
The fusion results of different fusion methods for the first set of CT and MRI images: (a), (b) Source images; fused images by (c) DWT, (d) GP, (e) PCA, (f) GF, (g) CT, (h) NSCT-1, (i) NSCT-2, and (j) the proposed method.

How to quantify the dependencies between NSCT coefficients by a statistical model is a subject worthy of study. Inspired by the earlier statistical model of MSD coefficients of image [23–26], in which the PDFs of coefficients across scales and within scale are nicely fitted by the GGD function, we fit the distribution characteristics by the same way and calculate the dependencies of the NSCT coefficients.  Figure 5 provides four PDFs of the NSCT coefficients together with the curves of the fitted GGDs (as shown purple curves). It can be seen that these fitted curves are very close to the actual case. Therefore, the statistical model can be applied to describe the spatial distribution characteristics of the NSCT coefficients.

### 3.3. Statistics of the NSCT Coefficients

The GGD model has been extensively applied to describe the marginal density of subband coefficients due to its flexible parametric form, which adapts to a large family of distributions from super-Gaussian to sub-Gaussian. Accordingly, the approximation of the marginal density for the particular NSCT coefficient may be achieved by varying two parameters of the GGD, which is defined as

(1) $$\begin{array}{c} P\left(\;x;\alpha ,\beta \;\right)=\frac{\beta }{2\alpha \Gamma \left(1/\beta \right)}e^{-{\left(\left|x\right|/\alpha \right)}^{\beta }}, \end{array}$$

where Γ(·) is the Gamma function, *α* is the scale parameter (width of the PDF peak), and *β* is the shape parameter which tunes the decay rate of the density function. Normally, the parameters *α* and *β* are computed by the maximum likelihood (ML) estimator, which has shown to be the desired estimator [27]. As for each subband, the likelihood function of the sample *x* = (*x*_1_, *x*_2_,…, *x*_*n*_) is defined as

(2) $$\begin{array}{c} L\left(\;x;\alpha ,\beta \;\right)=\log\overset{n}{\underset{i=1}{\prod }}p\left(\;x_{i};\alpha ,\beta \;\right). \end{array}$$

In this case, *α* and *β* are parameters that need to be estimated. We can obtain the unique root by the likelihood equations below; here Ψ(·) denotes the digamma function:

(3) ∂Lx;α,β∂β−Lα+∑i=1Nβxiβα−βα=0,

(4) ∂Lx;α,β∂βLβ+LΨ1/ββ2−∑i=1Nxiαβlog⁡xiα=0.

Let *β* be fixed and *β* > 0; then (4) has the unique solution, which is the real and positive value:

(5) $$\begin{array}{c} \hat{\alpha }={\left(\;\frac{\beta }{N}\overset{N}{\underset{i=1}{\sum }}{\left|\;x_{i}\;\right|}^{\beta }\;\right)}^{1/\hat{\beta }}. \end{array}$$

Combining (4) and (5), the shape parameter *β* can be solved by the following transcendental equation:

(6) 1+Ψ1/β^β^−∑i=1Nxiβ^log⁡xi∑xiβ^+log⁡β^/N∑i=1Nxiβ^β^=0.

In (6), the determination of $\hat{\beta }$ can be effectively solved using Newton-Raphson iterative procedure [26, 28] and the algorithm is detailedly described in [22]. Therefore, with only two parameters, we can accurately characterize the marginal distribution of the NSCT coefficients.

### 3.4. The Dependency of Different NSCT Subbands

The KLD is a common and justified way of measuring the distance between two distributions. *D*_KL_(*P*||*Q*) is applied to describe the deficiency of using one distribution *q* to represent the true distribution *p*, which is generally used for comparing two related distributions. The KLD between two distributions for *P*, *Q*, the PDFs of which are, respectively, denoted as *p*(*X*; *θ*_1_), *p*(*X*; *θ*_2_), is defined as

(7) DKLP||QDpx;θ1||px;θ2=∫px;θ1log⁡px;θ1px;θ2dx,

where *θ*_1_ and *θ*_2_ are a set of estimated parameters. Given two GGD distributions of NSCT subbands, the similarity between two GGDs for NSCT subbands can be defined by the parameters *α* and *β*. Substitute (1) into (7) and after some manipulations, the KLD between two PDFs can be expressed as

(8) DKLP||QDKLp·;a1,β1||p·;α2,β2=log⁡β1α2Γ1/β2β2α1Γ1/β1+α1α2β2Γβ2+1/β1Γ1/β1−1β1.

However, there are some deficiencies with the KLD, which makes it less ideal. First, the KLD is asymmetric; that is, (*D*_KL_(*P*||*Q*)) is different from (*D*_KL_(*Q*||*P*)). Second, if *q*(*x*) = 0 and *p*(*x*) ≠ 0 for any *x*, then *D*_KL_(*P*||*Q*) is undefined. Third, the KLD does not offer any nice upper bounds [14]. On the other hand, the JSD has the characteristics of nonnegativity, finiteness, symmetry, and boundedness [15, 29]. So we use the symmetric JSD to measure the similarity between two NSCT subbands in this study. The JSD between GGDs is derived from the KLD; mathematically, it is defined as

(9) DJSP||QDJSQ||P=12DKLP||M+DKLQ||M,M=P+Q2.

## 4. The Proposed Image Fusion Technique

### 4.1. Fusion of the Low Frequency Coefficients

The low frequency subbands represent the approximation components of the source images. The simplest way to combine subband coefficients is the averaging method. However, this method easily leads to the low contrast and blurred result. To extract more useful information from the source images, for the low frequency coefficients, we employ the fusion rule based on two activity level measurements, which consists of the regional standard deviation and Shannon entropy. In principle, the local texture features of an image are related with the variation of the coefficients around neighborhood. On the other hand, the entropy indicates how much information an image contains. Thus, combining the two together can extract more complementary information present in the source images. The process is listed as follows:

(1) Computing the regional standard deviation *D*_*λ*_(*x*, *y*)

(10) Dλx,y=∑m∈M,n∈Nωm,n×Cλx+m,y+n−Sλx,y2.

(2) Calculating the normalized Shannon entropy

(11) $$\begin{array}{c} E_{\lambda }\left(\;x,y\;\right)=\frac{1}{\left|R\right|}\underset{i,j}{\sum }{\left(\;C_{0}^{\lambda }\left(\;i,j\;\right)\;\right)}^{2}\log{\left(\;C_{0}^{\lambda }\left(\;i,j\;\right)\;\right)}^{2}. \end{array}$$

(3) Computing the weights (*δ*_*λ*_, *ξ*_*λ*_) of the standard deviation *D*_*λ*_(*x*, *y*) and the information entropy *E*_*λ*_(*x*, *y*), respectively,

(12) δλ=Dλx,yαDAx,yα+DBx,yα;ξλ=Eλx,yEAx,y+EBx,y,

where the parameter *α* is a constant, which tunes the sharpness of fused image by adjusting the value of parameter; it is set to 1.2 in our experiment.

Let *C*_0_^*λ*^(*x*, *y*) denote the low frequency subband coefficient at location (*x*, *y*); *λ* is input image *A*, *B*. Finally, the fused image can be obtained by

(13) $$\begin{array}{c} C_{0}^{F}\left(\;x,y\;\right)=\underset{\lambda =A,B}{\sum }\left[\;\delta_{\lambda }C_{0}^{\lambda }\left(\;x,y\;\right)+\xi_{\lambda }C_{0}^{\lambda }\left(\;x,y\;\right)\;\right]. \end{array}$$

### 4.2. Fusion of the High Frequency Coefficients

High frequency subbands correspond to detailed information in these regions such as edges, lines, and corners. Because different imaging modalities contain redundant and complementary information of each other, the purpose of selection rule is mainly to capture salient information of the source images as much as possible. Maximum selection rule is not suitable for medical image fusion, because it works well on this premise that only an original image provides good pixel at each corresponding location; thus vast complementary information will be lost when it is used for MIF. To improve the fusion performance, for the high frequency subbands, we propose the fusion scheme based on weight maps which are determined by the saliency maps. According to the fact that there exist dependencies between the NSCT coefficients, the high frequency coefficients are first updated by utilizing the relationships between NSCT subbands and then combining together by using weight maps. The process is described as follows.

(*1) Updating of the High Frequency Subband Coefficients*. First, we calculate the horizontal dependency *jsd*_*l*,*θ*,*h*_ between coefficients with different directions at the same scale *l* as

(14) $$\begin{array}{c} {jsd}_{l,\theta ,h}\left(\;x,y\;\right)=\overset{K}{\underset{j=1,j\neq i}{\sum }}D_{JS}\left(\;C_{l,\theta_{i}}\left(\;x,y\;\right),C_{l,\theta_{j}}\left(\;x,y\;\right)\;\right), \end{array}$$

where *K* is the total of the subbands at the *l*th scale.

Then we calculate the vertical dependency *jsd*_*l*,*θ*,*v*_ between the specified subband's (for instance subband *i*) parents and children

(15) jsdl,θ,vx,y=∑j=1KDJSCl,θix,y,Cl−1,θjx,y+DJSCl,θix,y,Cl+1,θjx,y.

Further, the horizontal and vertical dependency components are normalized, respectively,

(16) jsdl,θ,hx,y=jsdl,θ,hx,yjsdl,θ,hx,y+jsdl,θ,vx,y,jsdl,θ,vx,y=jsdl,θ,vx,yjsdl,θ,hx,y+jsdl,θ,vx,y.

Finally, the high frequency NSCT coefficients are revised as

(17) Cl,θx,y=Cl,θx,y1+jsdl,θ,hx,y2+jsdl,θ,vx,y2.

(*2) Construction of Weight Maps*. Weight maps are derived from the saliency maps, which describe each pixel by the saliency level of salient information. We apply Gaussian filter to each high pass subband, which tends to assign a high weight value to important elements such as edges and corners. A saliency map is constructed by the local average of the absolute value of the filter response

(18) $$\begin{array}{c} S_{l,\theta }\left(\;x,y\;\right)=\left|\;C_{l,\theta }\left(\;x,y\;\right)\;\right|*g_{r_{g},\theta_{g}}\left(\;x,y\;\right), \end{array}$$

where *g*(·) is a Gaussian low pass filter, whose size is (2*r*_*g*_ + 1)×(2*r*_*g*_ + 1), and the parameters *r*_*g*_ and *θ*_*g*_ are set to 5. Next, the weight maps are determined by comparison of the saliency maps (*S*_*l*,*θ*_^*n*^(*x*, *y*), *n* ∈ [*A*, *B*])

(19) Wl,θnx,y=1if  Sl,θnx,y=max⁡Sl,θAx,y,Sl,θBx,y0otherwise.

Finally, the fused subband coefficients *C*_*l*,*θ*_^*F*^(*x*, *y*) can be obtained by the weighted summation

(20) Cl,θFx,y=Wl,θAx,yCl,θAx,y+Wl,θBx,yCl,θBx,y.

## 5. Experimental Results and Discussion

Five different data sets of human brain images are used and the source images consist of two different modalities, including CT/MRI, MRI/PET, and MRI/SPECT images. All the images have the size of 256 × 256 pixels, which have been registered by some kind of registration method as [30]. To verify the effectiveness and applicability of the proposed fusion scheme, the results produced by the proposed method are, respectively, compared with results of other state-of-the-art schemes, such as discrete wavelet transform (DWT) [1], gradient pyramid (GP) [31], principal component analysis (PCA) [32], Intensity, Hue, and Saturation color model (IHS) [33], guided filtering (GF) [34], the contourlet transform (CT) [3], NSCT, the shearlet transform (ST) [35], and the nonsubsampled shearlet transform (NSST) based methods. For simplicity, MIF method [12] based on pulse-coupled neural network and modified spatial frequency in NSCT domain is denoted as NSCT-1. NSCT based MIF method in the scheme [36] is denoted as NSCT-2. The fusion method [37] based on neighborhood characteristic and regionalization in NSCT domain and NSCT based MIF method [10] in *l*, *α*, *β* color space are denoted as NSCT-3 and NSCT-4, respectively. Accordingly, NSST based MIF method using GGD model [21] is termed as NSST-1. NSST based fusion scheme of the literature [38] and MIF method [39] by utilizing the features in NSST domain are termed as NSST-2 and NSST-3, respectively. NSST based statistical MIF method [20] using HMT model is termed as NSST-4. For the NSCT and NSST methods, we adopt the average-maximum fusion scheme; namely, the low frequency coefficients are fused by the average of the corresponding coefficients and the high frequency coefficients are fused by using absolute maximum. For all MSD methods, the original images are all decomposed into 4 levels with the number of the directions 2, 2, 3, 3. Additionally, the quantitative comparison based on five image fusion quality metrics is also employed to demonstrate the fusion performance of different methods.

### 5.1. Experiments on CT-MRI Image Fusion

**Figure 6:**
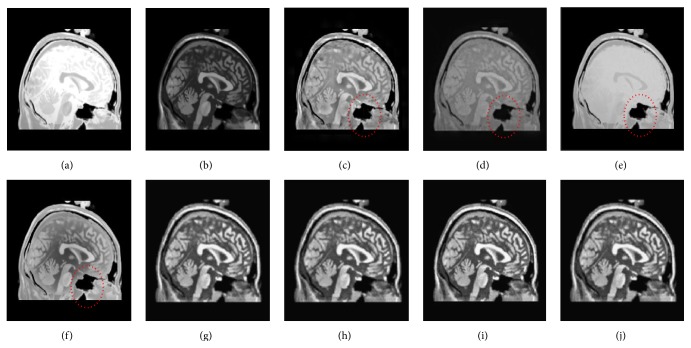
The fusion results of different fusion methods for the second set of CT and MRI images: (a), (b) Source images; fused images by (c) DWT, (d) GP, (e) PCA, (f) GF, (g) CT, (h) NSCT-1, (i) NSCT-2, and (j) the proposed method.

Figure 5 shows a fusion experiment of CT and MRI image. It can be seen that PCA based method gives poor result relative to the other algorithms, in which the bone structure of original CT image is almost invisible. For the GP based method, the final image is darker and has lower contrast, some detail information is unclear. The results from Figures 5(c), 5(f), 5(g), 5(h), and 5(i) have some improvement to various degrees and produce better visual effect on bone structures; however, the details of the soft tissue regions from these methods still retain unsharpness. By contrast, the proposed method can well preserve the detailed features of the original images without producing visible artifacts.  Figure 6 is another example of CT and MRI image fusion. As seen from Figures 6(c), 6(d), 6(e), and 6(f), their results have low contrast and lose a lot of details. What is worse, there are undesirable artifacts on the edges of these final images (see regions labeled by the red ellipses in Figure 6). Accordingly, CT based method, other NSCT based methods, and the proposed method provide better visual effects with good contrast; the abundant information of the source images can be successfully transferred to the fused image. Both tests imply that the proposed method is suitable for fusion of CT and MRI images.

### 5.2. Experiments on MRI-PET and MRI-SPECT Image Fusion

**Figure 7:**
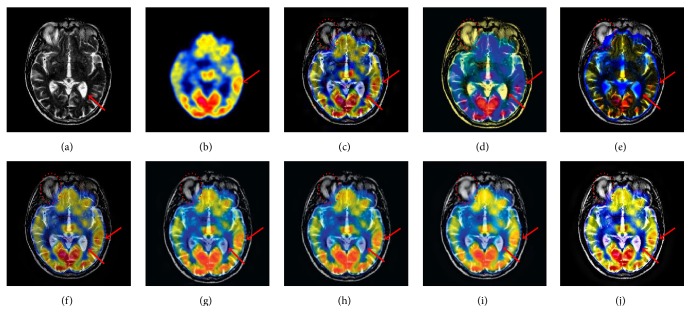
The fusion results of different fusion methods for the MRI and PET images: (a), (b) Source images; fused images by (c) DWT, (d) PCA, (e) IHS, (f) GP, (g) CT, (h) NSST, (i) NSST-1, and (j) the proposed method.

In this section, a case of MRI and PET fusion for a 70-year-old man affected with Alzheimer's disease is shown. In Figure 7, the source MRI image shows that the hemispheric sulci is widened and more prominent in the parietal lobes; the corresponding PET shows that regional cerebral metabolism is abnormal and hypometabolism heavily happens in anterior temporal and posterior parietal regions; meanwhile the right hemisphere is slightly more affected than the left. Here, the proposed method is compared with other seven fusion schemes. Obviously, the results of Figures 7(d) and 7(e) have disadvantages with serious color distortion and low contrast. Although the results of Figures 7(c) and 7(f) improve the fusion performance to some extent, they are not saturated in brightness, so that some parts are unidentifiable. Additionally, for the contrast and color fidelity, the results from Figures 7(g), 7(h), and 7(i) have better fusion performance than these methods mentioned above, but the structural information of the MRI image is not successfully transferred to fused images. Through the comparison of these fused results, it is found that the proposed method can well extract the structural and functional information from the source images and fuse them with much less information distortion. As illustrated in these regions highlighted by red arrows and ellipses in Figure 7, the proposed method well preserves complementary information of different modal medical images and achieves the best visual effect in terms of contrast, clarity, and color fidelity.

**Figure 8:**
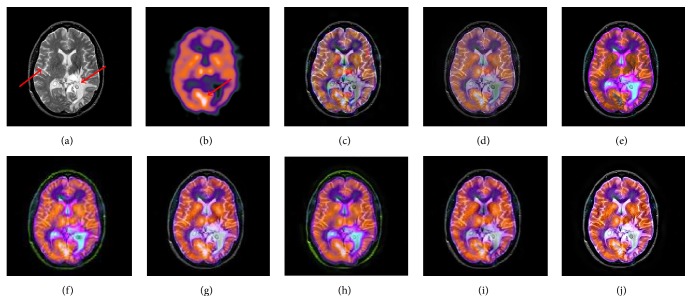
The fusion results of different fusion methods for the first set of MRI and SPECT images: (a), (b) Source images; fused images by (c) DWT, (d) GP, (e) IHS, (f) NSST-2, (g) NSST-3, (h) NSCT-3, (i) NSCT-4, and (j) the proposed method.

**Figure 9:**
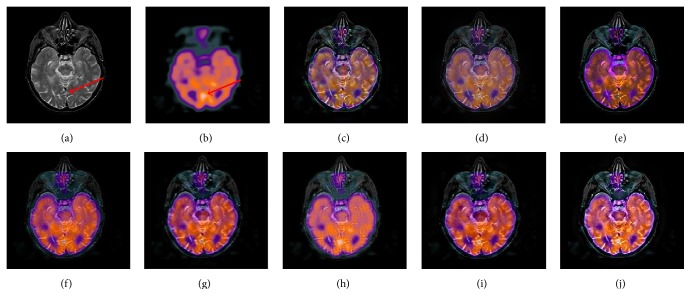
The fusion results of different fusion methods for the second set of MRI and SPECT images: (a), (b) Source images; fused images by (c) DWT, (d) GP, (e) IHS, (f) ST, (g) CT, (h) NSST-4, (i) NSCT, and (j) the proposed method.

Figure 8 is a fusion experiment of MRI and SPECT images. The source MRI image demonstrates that tumors are located in the left temporal region, as shown in the high signal intensity region (the white region labeled by the right red arrow in Figure 8(a)). From Figures 8(e), 8(f), and 8(h), the results produced by IHS based methods are distinctly color distortion in the lesion region. The results produced by the DWT and GP based methods cannot well inherit PET image's functional information and produce the low contrast images (see Figures 8(c) and 8(d)). By comparison, Figures 8(g) and 8(i) gain better results in terms of contrast and color fidelity. However, for the spatial details, the fused result obtained by the proposed method is more close to the original MRI image (see the region labeled by the left red arrow), and the spectral features are also natural.  Figure 9 provides another example of MRI and SPECT image fusion. In this test, the proposed method is specifically compared with the typical schemes [20], which is the MIF method based on the statistical dependencies between coefficients in NSST domain. From all the fused results, it is easily observed that the proposed method not only inherits the salient information existing in both the original images but also hardly causes the problem of color distortion. Through the above examples, it can be seen that the proposed method can be extended to combine the anatomical and functional medical images and achieves good visual effects.

### 5.3. Objective Evaluation and Analysis

In addition to the visual analysis, five fusion quality metrics, namely, mutual information (MI) [40], entropy (EN) [41], spatial frequency (SF), *Q* ^*AB*/*F*^ [42], and the uniform intensity distribution (UID) [43], are employed to test the validity of the proposed method. They reflect the fusion performance from clarity, contrast, color distortions, and the amount of information. MI, as an information measure for evaluating image fusion performance, represents how much information is obtained from the source images. The higher value of EN shows the fused image has more information contents and the higher value of SF indicates the final image is clearer. The index *Q* ^*AB*/*F*^ measures the amount of information transferred from source images to the fused image, and the UID is used for the description of uniform intensity and color distribution and the higher UID means better color information.

The quantitative comparisons are listed in Tables 1, 2, and 3. It can be seen that, for all the indices, the proposed method has a stable performance (most of values rank the first and only a few rank the second). It shows that the objective results based on these quality metrics also coincide with the subjective visual perception. Particularly, for the MI values of all tests, the proposed scheme all gets the largest value. It is confirmed that the proposed statistical model can transform more detailed information from the source images into the final image by exploiting dependencies between the NSCT coefficients. Therefore, it can be concluded that the proposed method is effective and is suitable for medical image fusion.

### 5.4. Computational Complexity Analysis

To investigate the computational complexity of different schemes, we record the running time of different fusion algorithms used in Figure 5 (see Table 4). All the tests are implemented by Matlab 2014a on a PC with double Intel core i7-3770k CPU @3.5 GHz, 8 GB RAM. As shown in Table 4, among all fusion methods, the consumption time of PCA based algorithm is the lowest, the reason of which is that it does not involve multiscale decomposition. Additionally, DWT, GP, GF, and CT based methods have also low time consumption (less than 0.08 s). However, their performance is poor. Relatively, three methods based on NSCT are slower, which is also a common problem of the algorithms based on NSCT. Due to using the complex neural network and mechanism of NSCT, NSCT-1 needs the most time (about 34 s). The proposed method and NSCT-2 consume the similar time (17.13 s and 15.29 s). Actually, the decomposition and reverse construction almost cost 9/10 of the total time. Without exception, the proposed method increases the cost of computational complexity with utilizing NSCT tool, yet it achieves the better effect than previous methods.

## 6. Conclusions

In this paper, we propose a novel NSCT based statistical multimodal medical image fusion method, which utilizes GGD to fit nicely marginal distributions of the high frequency coefficients and accurately measures the similarity between two NSCT subbands by the JSD of two GGDs. The proposed fusion rules make full use of the dependencies between the coefficients and transfer them to the final image. Experimental results demonstrate that the proposed algorithm can effectively extract the salient information from the source images and well combine them. Note that the fusion methods based on NSCT lack the competitive advantage in time consumption because of multilevel decomposition and reconstruction process. Fast image multiscale transform tool is the subject of future research.

## Figures and Tables

**Table 1:** Objective evaluation results of the four different metrics for CT and MRI images.

	Index	DWT	GP	PCA	GF	CT	NSCT-1	NSCT-2	Proposed
Figure 5	MI	1.274	1.279	1.365	1.279	1.324	1.450	1.490	1.891
	EN	5.008	4.843	4.050	5.107	5.183	4.978	5.371	5.675
	SF	33.22	31.08	20.52	29.83	27.67	23.52	33.41	34.86
	*Q* ^*AB*/*F*^	0.509	0.577	0.369	0.592	0.592	0.568	0.593	0.596

Figure 6	MI	3.077	2.521	3.663	2.729	3.831	3.916	3.949	3.962
	EN	5.125	4.851	4.340	5.578	6.300	6.414	6.473	6.469
	SF	22.79	19.84	19.04	20.29	29.87	30.15	30.27	31.41
	*Q* ^*AB*/*F*^	0.618	0.694	0.665	0.682	0.682	0.694	0.699	0.703

**Table 2:** Objective evaluation results of the five different metrics for MRI and PET images.

	Index	DWT	PCA	IHS	GP	CT	NSST	NSST-1	Proposed
Figure 7	MI	2.278	2.841	2.656	2.280	2.108	2.174	2.513	2.853
	EN	4.573	3.707	3.125	4.561	4.242	4.148	5.080	4.768
	SF	30.21	31.14	16.03	30.12	31.30	31.15	32.21	33.73
	*Q* ^*AB*/*F*^	0.525	0.498	0.483	0.526	0.551	0.584	0.617	0.607
	UID	0.752	0.737	0.725	0.753	0.789	0.797	0.836	0.814

**Table 3:** Objective evaluation results of the five metrics for MRI and SPECT images.

	Index	DWT	GP	IHS	NSST-2	NSST-3	NSCT-3	NSCT-4	Proposed
Figure 8	MI	2.167	2.028	2.255	2.399	2.688	2.376	2.520	2.695
	EN	4.424	3.973	4.105	4.870	4.507	3.533	4.499	4.531
	SF	31.89	29.57	35.06	35.28	40.17	34.79	39.78	41.20
	*Q* ^*AB*/*F*^	0.487	0.481	0.512	0.499	0.556	0.507	0.536	0.576
	UID	0.802	0.812	0.711	0.724	0.817	0.765	0.813	0.826

	Index	DWT	GP	IHS	ST	CT	NSST-4	NSCT	Proposed

Figure 9	MI	2.485	2.113	2.872	2.480	2.532	3.076	2.784	3.083
	EN	4.277	4.050	4.202	4.561	4.426	4.594	4.202	4.668
	SF	31.24	29.48	31.26	35.47	40.69	39.45	40.87	41.76
	*Q* ^*AB*/*F*^	0.518	0.506	0.502	0.537	0.536	0.538	0.519	0.540
	UID	0.793	0.679	0.714	0.783	0.799	0.806	0.801	0.817

**Table 4:** Computational complexity comparison of different fusion methods for CT-MRI dataset shown in Figure 5.

Methods	DWT	GP	PCA	GF	CT	NSCT-1	NSCT-2	Proposed
Running time (s)	0.021	0.019	0.003	0.075	1.976	33.79	15.29	17.13
